# Near-death experiences in non-life-threatening events and coma of different etiologies

**DOI:** 10.3389/fnhum.2014.00203

**Published:** 2014-05-27

**Authors:** Vanessa Charland-Verville, Jean-Pierre Jourdan, Marie Thonnard, Didier Ledoux, Anne-Francoise Donneau, Etienne Quertemont, Steven Laureys

**Affiliations:** ^1^Coma Science Group, Cyclotron Research Center and Neurology Department, University and University Hospital of LiègeLiège, Belgium; ^2^International Association For Near Death StudiesOraison, France; ^3^Department of Public Health, University of LiègeLiège, Belgium; ^4^Department of Psychology, Cognitive and Behavioral Neurosciences Center, University of LiègeLiège, Belgium

**Keywords:** Near-death experiences, Greyson NDE scale, coma, cardiac arrest, traumatic brain injury, memory, non-life threatening events

## Abstract

**Background:** Near death experiences (NDEs) are increasingly being reported as a clearly identifiable physiological and psychological reality of clinical significance. However, the definition and causes of the phenomenon as well as the identification of NDE experiencers is still a matter of debate. To date, the most widely used standardized tool to identify and characterize NDEs in research is the Greyson NDE scale. Using this scale, retrospective and prospective studies have been trying to estimate their incidence in various populations but few studies have attempted to associate the experiences' intensity and content to etiology.

**Methods:** This retrospective investigation assessed the intensity and the most frequently recounted features of self-reported NDEs after a non-life-threatening event (i.e., “NDE-like” experience) or after a pathological coma (i.e., “real NDE”) and according to the etiology of the acute brain insult. We also compared our retrospectively acquired data in anoxic coma with historical data from the published literature on prospective post-anoxic studies using the Greyson NDE scale.

**Results:** From our 190 reports who met the criteria for NDE (i.e., Greyson NDE scale total score >7/32), intensity (i.e., Greyson NDE scale total score) and content (i.e., Greyson NDE scale features) did not differ between “NDE-like” (*n* = 50) and “real NDE” (*n* = 140) groups, nor within the “real NDE” group depending on the cause of coma (anoxic/traumatic/other). The most frequently reported feature was peacefulness (89–93%). Only 2 patients (1%) recounted a negative experience. The overall NDE core features' frequencies were higher in our retrospective anoxic cohort when compared to historical published prospective data.

**Conclusions:** It appears that “real NDEs” after coma of different etiologies are similar to “NDE-like” experiences occurring after non-life threatening events. Subjects reporting NDEs retrospectively tend to have experienced a different content compared to the prospective experiencers.

## Introduction

The expression “Near-Death Experience” (NDE) was first formulated in the nineteenth century when a Swiss geologist collected testimonies from his fellow climbers and himself after making a fall in the Alps (Heim, 1891). Some decades later, Moody (1975) popularized the expression through his best seller “Life after life” in which he defined NDEs as “any conscious perceptual experience occurring in individuals pronounced clinically dead or who came very close to physical death” and drew a list of the most frequently recounted features by individuals who had survived near-fatal incidents. These NDE features included: overwhelming feelings of peacefulness and well-being; painlessness; the impression of being located outside one's physical body; the impression of drifting through a dark environment that looks like a tunnel; the sight of a brilliant light and the feeling of being surrounded by it; the impression of discovering another world described as idyllic; the feeling of a close presence; the encounter and communication with spirits or deceased relatives or experiencing a “life review” (Moody, 1975). More recently, NDEs have been defined as a “profound psychological event including transcendental and mystical elements, typically occurring to individuals close to death or in situations of intense physical or emotional danger” (Greyson, 2000).

Empirical studies of NDEs have mostly been conducted in patients with life threatening situations such as cardiac arrest (Parnia et al., 2001; van Lommel et al., 2001; Schwaninger et al., 2002; Greyson, 2003a; French, 2005; Klemenc-Ketis et al., 2010) or (albeit more rarely) in patients with severe traumatic brain injury (Hou et al., 2013). To the best of our knowledge, no study has formally compared the influence of the cause of coma to the intensity or content of the NDE. Unlike these “real NDEs” associated with “real” closeness to death or coma, possible NDEs or “NDE-like” experiences have also been reported in situations where there was no genuine threat to the individuals' life. Only a few studies have assessed “NDE-like” phenomena in non-life threatening situations (Gabbard et al., 1981; Owens et al., 1990; Gabbard and Twemlow, 1991; Kelly, 2001; Facco and Agrillo, 2012). Such accounts have also been reported in epileptic patients (Hoepner et al., 2013), syncope (Lempert et al., 1994), intense grief and anxiety (Kelly, 2001), Cotard's syndrome (McKay and Cipolotti, 2007; Charland-Verville et al., 2013) and during meditative state (Beauregard et al., 2009). It remains unclear whether NDEs occurring in life-threatening or non-life threatening situations differ in intensity or core features. In addition to the ill-described relation between the NDE and the precipitating factor, the reliability of NDEs accounts also remains controversial (Martens, 1994; French, 2001). For instance, it seems that fewer cases of NDEs are recounted by individuals interviewed prospectively than when the interviews are conducted retrospectively (Mobbs and Watt, 2011). On the other hand, it has been shown that reports of NDEs were not modified over time even 20 years after the original account (Greyson, 2007).

Using the Greyson NDE scale (Greyson, 1983), the present retrospective study aimed at (1) exploring the NDE intensity and content in “NDE-like” accounts following non-life-threatening events vs. “real NDE” following coma; (2) comparing the “real NDE” characteristics according to the etiology of the brain damage (anoxic, traumatic or other) and (3) comparing our retrospectively obtained data in anoxic coma to historical previously published prospectively collected post-anoxic NDEs.

## Materials and methods

Participants were recruited via the website, publications and appearances in local media of the International Association for Near-Death Studies (IANDS France and IANDS Flanders) and the Coma Science Group (University of Liège, Belgium). Written, email or telephone completion of an anonymous questionnaire was voluntary and taken as consent for participation in the survey. The study was approved by the ethics committee of the Faculty of Medicine of the University of Liège. The questionnaire included items about demographic (age at NDE, gender) and clinical data (time since NDE, presence of life threatening event, presence of acute coma (i.e., a period of unconsciousness >1 h; Posner et al., 2007)), etiology of coma (anoxic/traumatic/other) and a standardized characterization of the NDE using the Greyson NDE scale (Greyson, 1983). The Greyson NDE scale is a validated (Lange et al., 2004) 16-item multiple-choice tool used to quantify the intensity of the NDE (i.e., total score ranging from 0 to 32) and to assess core content components of 16 NDE features. For each item, the scores are arranged on an ordinal scale ranging from 0 to 2 (i.e., 0 = “not present,” 1 = “mildly or ambiguously present,” and 2 = “definitively present”; Greyson, 1983; Lange et al., 2004). For statistical analyses, a feature was considered present when participants scored an item as 1 or 2 (Greyson, 1983, 2003a). Participants whose experience did not meet the accepted criteria of NDE (i.e., Greyson total score <7; Greyson, 1983) were excluded from the present analysis.

First, we verified that “NDE-like” and “real NDE” groups and the 3 coma etiologies of the “real NDE” group (i.e., anoxic/traumatic/other) were matched for gender, age at NDE, and interval since NDE. A Pearson's chi square test with contingency tables was performed to compare the gender ratios between the “NDE-like” and “real NDE” groups as well as between the 3 coma etiologies (i.e., anoxic/traumatic/other) of the “real NDE” group. A Student's *t*-test was performed to compare age at NDE and time since NDE between the “NDE-like” and “real NDE” groups and a One-Way ANOVA was performed to compare the age at NDE and the time since NDE within the coma 3 etiologies of the “real NDE” group. Next, we looked for differences in NDE overall intensity (i.e., total NDE scale's scores) between “NDE-like” and “real NDE” groups' using Student's *t*-testing. A Pearson's chi square test with contingency tables was performed to assess for possible discrepancies between the reported features frequencies in “NDE-like” and “real NDE” groups. A One-Way ANOVA was performed in order to test for differences in intensity within the “real NDE” group depending on the etiology of coma. A Pearson's chi square test with contingency tables was performed to assess for possible discrepancies between the reported features frequencies according to the coma etiology. Finally, the present retrospective data in anoxic coma were compared with a historical dataset of prospective data taken from the published literature on NDE after anoxic coma (Parnia et al., 2001; Schwaninger et al., 2002; Greyson, 2003a). For each feature of the Greyson NDE scale, the proportions of positive answers between retrospective and prospective studies were compared by the classical chi-squared test for contingency tables or by the Fisher exact test. Further, the overall comparison between the two study groups was made by the Generalized Estimating Equations Approach (GEE), which accounts for percentage variability within NDE features.

## Results

Results were considered to be significant at the 5% critical level (*p* < 0.05) and were expressed as mean ± standard deviation (SD) for quantitative variables and as counts and proportions (%) for categorical variables. Data analysis was carried out using SAS (version 9.3 for Windows) statistical package. Out of the 215 self-reported NDEs, 25 did not meet the criteria for NDE (i.e., NDE scale total score <7/32) (Greyson, 1983) and were excluded from the current study. Table 1 shows the demographic characteristics of the different groups constituting the retrospective study cohort (*n* = 190; 104 females (55%); age at NDE 32 ± 14 year; time since NDE 24 ± 15 year). The “NDE-like” group (*n* = 50) included NDEs occurring following a non-life-threatening event such as during sleep (*n* = 13), syncope (*n* = 11), meditation (*n* = 5), drugs and alcohol consumption (*n* = 3), or other non-life threatening situations (*n* = 18). The “real NDE” group (*n* = 140) was divided according to the etiology of the coma: “anoxic” (e.g., cardiac arrest, near-drowning, *n* = 45); “traumatic” (e.g., motor vehicle accident, falls, *n* = 30) and “other” (i.e., non-traumatic events such as an exacerbation of on-going illness, complication during surgery, *n* = 65). There were no significant differences in gender, age at time of NDE and interval since NDE between the “NDE-like” and “real NDE” groups and within the “real NDE” groups depending on etiology.

**Table 1:** **NDErs demographic characteristics (*N* = 190)**.

**Demographics**	**“NDE-like” *n* = 50**	**“Real NDE” *n* = 140**	***p***	**“Real NDE” etiologies**			***p***
				**Anoxic *n* = 45**	**Traumatic *n* = 30**	**Other *n* = 65**	
Gender—female	25 (50%)	79 (57%)	0.43	20 (44%)	15 (50%)	44 (68%)	0.20
Age at NDE	31 ± 11	32 ± 15	0.76	35 ± 16	29 ± 11	31 ± 17	0.26
(Mean in years ± *SD*, range)	4–60	3–76		8–64	6–50	3–76	
Time since NDE	23 ± 16	24 ± 15	0.82	20 ± 14	26 ± 11	25 ± 17	0.10
(Mean in years ± *SD*, range)	0.13–66	0.15–75		0.15–50	3–43	1–75	

Intensity (i.e., Greyson NDE scale total score) and content (i.e., NDE scale features) of reported NDEs did not differ between “NDE-like” and “real NDE” groups nor within the “real NDE” group depending on the cause of coma (anoxic/traumatic/other) (see Table 2). For all study groups, the most frequently reported core NDE feature was the feeling of peacefulness for all study groups (frequency range: 89–93%). Only 18/190 (10%) did not experience positive emotions such as peacefulness, happiness or joy; 2 of whom explicitly recounted a negative experience (both following a life threatening event; 2 females, aged 31 and 41 who were comatose due to a complication after childbirth and surgery, respectively). Finally, the overall NDE core features' frequencies were significantly higher in our retrospective anoxic cohort (*n* = 45; 20 females 44%) when compared to historical published prospective data (Parnia et al., 2001; Schwaninger et al., 2002; Greyson, 2003a) obtained after anoxic coma (*p* < 0.0001). An altered time perception, the feeling of harmony and unity, the sudden understanding of everything, heightened senses were more frequently reported in the current retrospective dataset on post-anoxic NDE while encounters with deceased or religious spirits were more frequently reported in previous prospective studies on post-anoxic NDE (see Table 3).

**Table 2:** **NDE intensity (Greyson Scale total score) and content (core features) reported in “NDE-like” and “real NDE” groups–by decreasing order of frequency according to the “real NDE” group (significance level *p* < 0.05)**.

**NDE Scale features**	**“NDE-like” *n* = 50 (%)**	**“Real NDE” *n* = 140 (%)**	***p***	**“Real NDE” etiologies**			***p***
				**Anoxia *n* = 45 (%)**	**Trauma *n* = 30 (%)**	**Other *n* = 65 (%)**	
“Did you have a feeling of peace or pleasantness?”	45 (90)	127 (91)	0.88	40 (89)	28 (93)	59 (91)	0.81
“Did you feel separated from your body?”	37 (74)	111 (79)	0.44	36 (80)	24 (80)	51 (79)	0.98
“Did you see, or feel surrounded by, a brilliant light?”	42 (84)	106 (76)	0.23	32 (71)	20 (67)	54 (83)	0.15
“Did time seem to speed up or slow down?”	41 (82)	105 (75)	0.31	35 (78)	24 (80)	46 (71)	0.55
“Did you seem to enter some other, unearthly world?”	35 (70)	101 (72)	0.77	33 (73)	21 (70)	47 (72)	0.95
“Did you have a feeling of joy?”	37 (74)	98 (70)	0.59	32 (71)	18 (60)	48 (74)	0.38
“Did you feel a sense of harmony or unity with the universe?”	39 (78)	96 (69)	0.21	33 (73)	21 (70)	42 (65)	0.61
“Did you come to a border or point of no return?”	31 (62)	86 (61)	0.94	22 (49)	20 (67)	44 (68)	0.11
“Were your senses more vivid than usual?”	34 (68)	84 (60)	0.32	25 (56)	16 (53)	43 (66)	0.38
“Did you suddenly seem to understand everything?”	25 (50)	76 (54)	0.60	26 (58)	20 (67)	30 (46)	0.15
“Did you seem to encounter a mystical being or presence, or hear an unidentifiable voice?”	29 (58)	71 (51)	0.38	20 (44)	12 (40)	39 (60)	0.12
“Were your thoughts speeded up?”	20 (40)	62 (44)	0.60	23 (51)	15 (50)	24 (37)	0.26
“Did you see deceased or religious spirits?”	17 (34)	54 (39)	0.57	12 (27)	9 (30)	33 (51)	0.063
“Did you seem to be aware of things going on elsewhere, as if by ESP?”	14 (28)	47 (34)	0.50	10 (22)	9 (30)	28 (43)	0.067
“Did scenes from your past come back to you?”	9 (18)	37 (26)	0.23	11 (24)	11 (37)	15 (23)	0.35
“Did scenes from the future come to you?”	9 (18)	26 (19)	0.93	8 (18)	5 (17)	13 (20)	0.92
Total score (mean ± *SD*, range)	17 ± 7, 7–30	16 ± 6, 7–30	0.10	15 ± 6, 7–28	16 ± 6, 7–26	16 ± 6, 7–30	0.29

**Table 3:** **Retrospective and prospective anoxic studies' demographics and Greyson NDE scale core features (significance level *p* < 0.05)**.

**Study's demographics**	**Current data retrospective**	**Total prospective**	**Greyson (2003a) prospective**	**Schwaninger et al. (2002) prospective**	**Parnia et al. (2001) prospective**	***p***	**Odds ratio**	**95% interval**
Number of subjects	*N* = 45	*N* = 42	*N* = 27	*N* = 11	*N* = 4			
Age at NDE (years)	35 ± 16, 8–64		56 ± 13	53, 23–84	>18			
Gender	20 females (44%)	19 females (45%)	10 females (37%)	7 females (64%)	2 females (50%)		0.97	0.42–2.26
Etiology	Anoxic/hypoxic		Anoxic/hypoxic	Anoxic/hypoxic	Anoxic/hypoxic			
Time since insult (mean, *SD*, range)	20 ± 14 year 0.15–50 year		“4 days on average”	“2–3 days on average”	“<7 days”			
NDE Scale total score (*SD*)	15 ± 6		13 ± 6	N/D	11 ± 2	0.52		
Peacefulness	40 (89)	37 (88)	23 (85)	11 (100)	3 (75)	0.99	1.08	0.29–4.04
Out-of-body experience	36 (80)	31 (74)	19 (70)	10 (90)	2 (50)	0.49	1.42	0.52–3.87
**Altered time perception**	**35 (78)**	**21 (50)**	**18 (67)**	**1 (9)**	**2 (50)**	**0.007**	**3.50**	**1.38–8.85**
Unearthly environment	33 (73)	25 (60)	17 (63)	6 (54)	2 (50)	0.17	1.87	0.76–4.62
**Harmony/unity**	**33 (73)**	**21 (50)**	**14 (52)**	**5 (45)**	**2 (50)**	**0.025**	**2.75**	**1.12–6.74**
Happiness/joy	32 (71)	23 (55)	18 (67)	2 (18)	3 (75)	0.11	2.03	0.84–4.93
Bright light	32 (71)	29 (69)	19 (70)	7 (63)	3 (75)	0.83	1.10	0.44–2.76
**Understanding**	**26 (58)**	**11 (26)**	**8 (30)**	**2 (18)**	**1 (25)**	**0.003**	**3.86**	**1.56–9.55**
**Heightened senses**	**25 (56)**	**12 (29)**	**4 (15)**	**6 (54)**	**2 (50)**	**0.011**	**3.13**	**1.28–7.62**
Speeded thoughts	23 (51)	13 (31)	12 (44)	1 (9)	0 (0)	0.056	2.33	0.97–5.61
Border/point of no return	22 (49)	20 (48)	11 (41)	5 (45)	4 (100)	0.91	1.05	0.45–2.44
Mystical being/presence	20 (44)	15 (36)	7 (26)	7 (63)	1 (25)	0.41	1.44	0.61–3.41
**Encounter with deceased/religious spirits**	**12 (27)**	**24 (57)**	**14 (52)**	**8 (72)**	**2 (50)**	**0.004**	**0.27**	**0.11–0.67**
Extrasensory perception	10 (22)	5 (12)	3 (11)	0 (0)	2 (50)	0.74	2.11	0.66–6.80
Life review	11 (24)	9 (21)	8 (30)	1 (9)	0 (0)	0.20	1.19	0.44–3.23
Precognitive visions	8 (18)	3 (7)	2 (7)	1 (9)	0 (0)	0.14	2.81	0.69–11.41

## Discussion

We here used the Greyson NDE scale (Greyson, 1983) to retrospectively assess the characteristics of 190 self-reported NDEs precipitated by a non-life threatening event or coma. The Greyson NDE scale is a widely used and validated tool to assess the intensity and content of NDEs (Greyson, 1990, 2003a,b; Parnia et al., 2001; Schwaninger et al., 2002; Nelson et al., 2006; Lai et al., 2007; Klemenc-Ketis et al., 2010; Hoepner et al., 2013; Hou et al., 2013) and, in contrast to the Weighted Core Experience Index (WCEI; Ring, 1980), provides a cut-off score permitting a standardized identification of NDE experiencers (NDErs). As for other retrospective studies on NDEs (Greyson, 1990; Lai et al., 2007), the interval between the age at study enrollment (mean age 56 ± 13 year) and the occurrence of the NDE (mean age 32 ± 15 year) was several decades (mean 24 ± 15 year). Our study sample did not show a significantly higher proportion of female NDErs. In the “real NDE” coma-survival group, the most frequently reported features (i.e., occurring >75%) include the feeling of peacefulness, out-of-body-experiences, seeing a bright light, alerted time perception. Conversely, precognitive visions (e.g., seeing the future) and the experience of life review were among the least frequently reported core features (i.e., occurring <30%). These results corroborate previous reports on NDE studies using the Greyson NDE scale (Greyson, 1990, 2003a; Parnia et al., 2001; Schwaninger et al., 2002; Nelson et al., 2006; Lai et al., 2007) or the WCEI (van Lommel et al., 2001). According to the etiology of coma, a recent prospective study reporting interview transcripts of post-traumatic coma survivors with a NDE revealed that the most frequently reported elements were the sight of an intense light, feelings of astonishment, pleasure and sense of helplessness (Hou et al., 2013). Although the well-being component is one of the most often reported features in classical NDEs, it is important to note that distressing or hellish experiences can also occur. In line with previous estimations (Lindley et al., 1981; Gallup and Proctor, 1982; Sabom, 1982; Ring, 1984), we recorded an incidence of 1.4% (2/140) of our “real NDE” group that reported to have had a negative NDE.

Some authors have tried to explain the phenomenology of NDE by diverse physiological explanations such as anoxic brain damage (Rodin, 1980; Blackmore, 1993; Greyson, 1998; Els et al., 2004; Woerlee, 2005; Ammermann et al., 2007), hypoxia (Lempert et al., 1994), hypercapnia (Klemenc-Ketis et al., 2010), abnormal temporal lobe dysfunctions (Blanke et al., 2002, 2004; Britton and Bootzin, 2004; Blanke and Mohr, 2005; Arzy et al., 2006; Hoepner et al., 2013), administration of sedatives (Cobcroft and Forsdick, 1993; Osterman et al., 2001; Lopez et al., 2006), or sleep abnormalities (Nelson et al., 2006, 2007). However, to the best of our knowledge, previous studies on NDE after coma have not aimed at identifying differences in NDE characteristics depending on the etiology (i.e., traumatic, non-traumatic anoxic or non-traumatic other acute brain insults) of the prolonged loss of consciousness. Despite our relatively large sample size, our analyses failed to show a significant difference on NDE intensity or content between these different causes of coma (matched for age, gender and interval since NDE). In the current study sample, we did not observe a significant difference in NDE intensity or core feature frequency when comparing “real NDE” after coma to “NDE-like” events occurring after non-life-threatening events. Some authors have argued that the strong belief or fear of dying might be the key determinant for triggering a NDE (Gabbard et al., 1981; Stevenson et al., 1989–1990; Gabbard and Twemlow, 1991) independently of the actual organic brain damage.

Features of a NDE occurring during situations that are not life threatening and that are not perceived as such like during sleep or a meditative state cannot be explained by the expectancy of an incoming death. Individuals put in apparently life-threatening situations (e.g., involved in various accidents or undergoing surgery) might think the worst could happen even though the real medical situations result in none or minor brain insults (Gabbard and Twemlow, 1991; Facco and Agrillo, 2012). Likewise, the first historical reports of NDE testimonies obtained from Alpine mountain climbers who had suffered a non-fatal fall also illustrate the role of psychological reactions to a perceived life-threatening event in the generation of NDEs (Heim, 1891). Owens et al. (1990) reported that both “NDE like” and “real NDEs” showed a comparable phenomenology including positive emotions, out-of-body experiences, tunnel-like perceptions and memory flashbacks. In contrast to our findings they reported that NDErs who had been in a life threatening condition (i.e., “real NDE”) tended to more frequently report seeing a bright light and experiencing enhanced cognitive functions (e.g., speeded thoughts and sense of understanding). Gabbard and Twemlow (1991), on the other hand, observed that individuals who self-reported a “close brush with death,” as compared to those who did not, experienced more out-of-body experiences. It is difficult to compare these findings with the present data because both previous studies did not employ a standardized scale for the characterization of the NDE. Using the Greyson NDE scale, Kelly (2001) reported that individuals who were close to death more often encountered deceased relatives during the NDE. Furthermore, the reports of those encounters were more frequent when the precipitating factor was a traumatic brain injury or a cardiac arrest than when the NDE was precipitated by a complication during childbirth or surgery (Kelly, 2001). The fact that we did not observe such differences in our current sample could be explained by the heterogeneity of the investigated samples' etiologies as well as the ill-defined nature of the non-life-threatening conditions.

**Figure 1:**
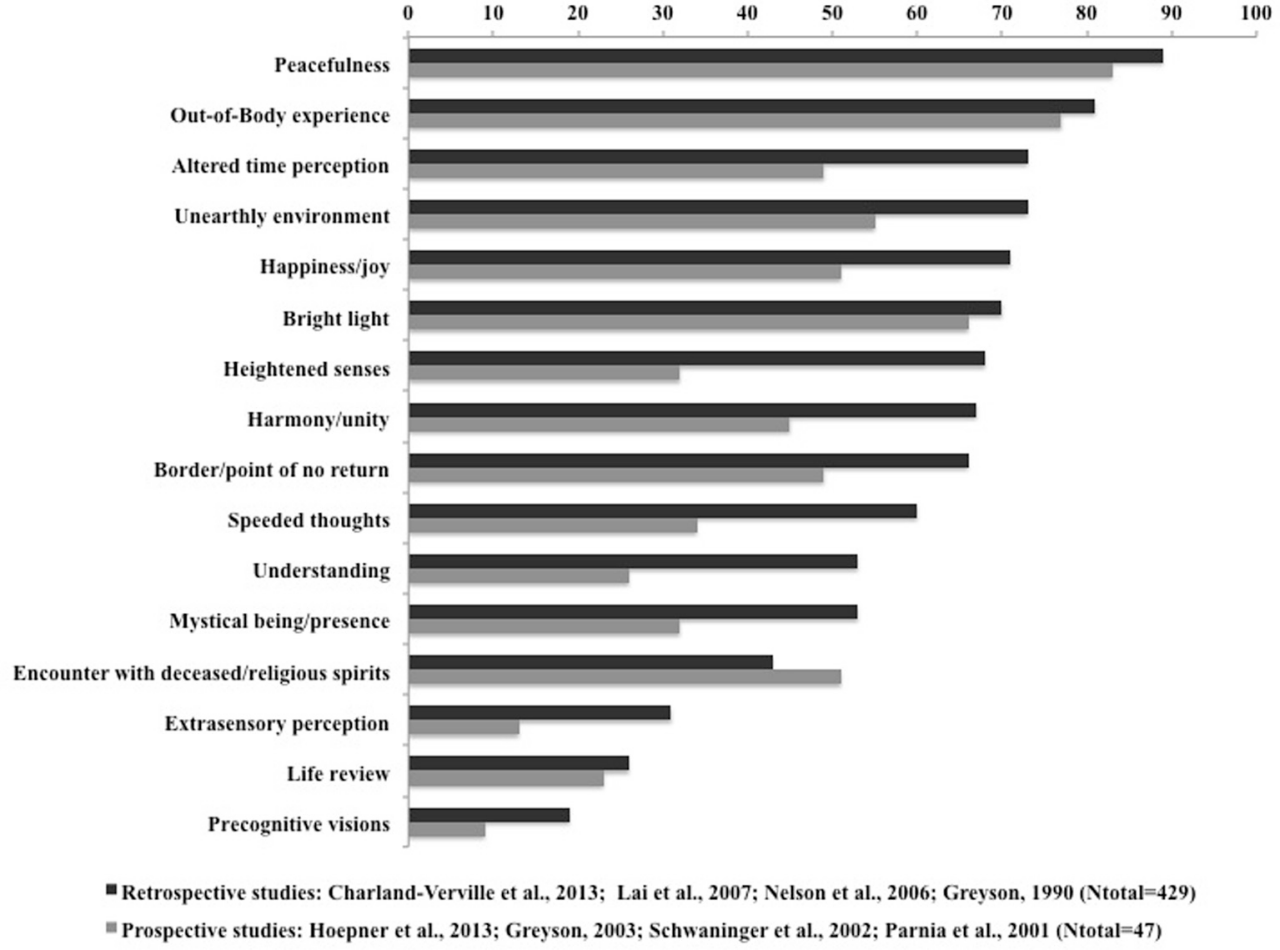
**Frequency of NDE features in retrospective and prospective studies (all etiologies) according to the Greyson NDE scale by decreasing order according to the retrospective data**.

It is important to stress that our study has a number of methodological limitations. Retrospective recruitment of self-reported NDEs may not represent a reliable sample of the NDErs population since they might have greater interest in and knowledge of NDEs. In addition to the probable sample-bias related to the recruitment of medically uncontrolled NDEs, the interval between the occurrence of the NDE and the age at study enrollment was several decades—similar to other retrospective surveys (Greyson, 1990; Lai et al., 2007). We therefore compared our retrospective data with prospectively obtained published datasets. In order to reduce the possible confound of heterogeneity in etiology and NDE characterization we have chosen to compare our retrospective anoxic coma group with results from published prospective NDE studies on anoxic coma also using the Greyson NDE scale (Parnia et al., 2001; Schwaninger et al., 2002; Greyson, 2003a). Our comparison identified a higher overall frequency of NDE core features occurrence in our retrospective sample (Table 3). Similarly, a recent review by Mobbs and Watt (2011) also points to a higher incidence of NDEs in retrospective as compared to prospective studies. These findings could be related to the claim of some authors that NDE reports might suffer from memory reconstructions (French, 2001; Martens, 1994). However, Greyson (2007) has reported that the memories of NDE core features did not modify over time. Moreover, our data suggest that encounters with deceased or religious spirits are more frequently reported *prospectively*. It should be stressed that any statistical comparison of different study designs (retro- vs. prospective) is methodologically problematic because of small sample sizes, high variability and sampling error. Consequently, it is difficult to assign appropriate weights reflecting the relative “value” of the information provided in each study. However, our findings on differences between retrospective and prospective post-anoxic NDE reports seem to be confirmed when extended to other etiologies as illustrated in Figure 1. A visual comparison of the weighted ratios of core NDE features calculated from four retrospective studies (Greyson, 1990; Nelson et al., 2006; Lai et al., 2007) (total *n* = 429; including the present dataset) and four prospective studies (Parnia et al., 2001; Schwaninger et al., 2002; Greyson, 2003a; Hoepner et al., 2013) (total *n* = 47), all using the Greyson NDE scale, illustrates that all items (with the exception of encounters with deceased or spirits) seem more frequently reported retrospectively.

It seems that NDEs cannot be explained solely by the closeness to death or by the etiology of the precipitating factor. The question whether the NDEs' extraordinary features can be fully explained by cerebral activity is still a matter of debate and a challenge awaiting the neuroscientific analysis of this phenomenon is to identify the neural correlates of such a physiologically real yet unexplained cognitive experience. Studying NDEs continues to represent a methodological challenge and investigators must rely on testimonies and indirect ways to understand the brain mechanisms associated with such an experience. When we compared our medically uncontrolled retrospectively obtained results to historical data from controlled prospective trials, several core features seemed to be more frequently reported when retrospectively collected (i.e., altered time perception, harmony, understanding and heightened senses). In line with our findings and as previously stressed (Facco and Agrillo, 2012), NDE research might benefit from the introduction of a new terminology to account for “NDE-like” experiences. In addition to the use of closed NDE questionnaires, which only leave restricted choices for describing the experience, future studies should employ statistical examination of freely expressed NDEs narratives using automated user-independent qualitative analyses of their content (Hou et al., 2013), taking into account the clinical data and study design.

## Author contributions

Vanessa Charland-Verville and Steven Laureys designed the study, wrote the manuscript and conducted the analyses. Vanessa Charland-Verville, Jean-Pierre Jourdan, Marie Thonnard, Steven Laureys, collected all data and contributed to the final manuscript. Anne-Francoise Donneau, Didier Ledoux and Etienne Quertemont contributed to the analyses and to the revision of the final manuscript. Jean-Pierre Jourdan and Steven Laureys provided conceptual input and contributed to the final manuscript. All authors approved the final version of the manuscript.

### Conflict of interest statement

The authors declare that the research was conducted in the absence of any commercial or financial relationships that could be construed as a potential conflict of interest.

## Abbreviations

Near-Death Experiences: NDEs
Near-Death Experiencers: NDErs
Weighted Core Experience Index: WCEI.

## References

[B1] Ammermann H. Kassubek J. Lotze M. Gut E. Kaps M. Schmidt J. (2007). MRI brain lesion patterns in patients in anoxia-induced vegetative state. J. Neurol. Sci., 260, 65–70 10.1016/j.jns.2007.03.026 17490686

[B2] Arzy S. Seeck M. Ortigue S. Spinelli L. Blanke O. (2006). Induction of an illusory shadow person. Nature 443, 287–287 10.1038/443287a 16988702

[B3] Beauregard M. Courtemanche J. Paquette V. (2009). Brain activity in near-death experiencers during a meditative state. Resuscitation 80, 1006–1010 10.1016/j.resuscitation.2009.05.006 19573975

[B4] Blackmore S. (1993). Dying to Live: Science and Near-Death Experience. London: Grafton

[B5] Blanke O. Landis T. Spinelli L. Seeck M. (2004). Out-of-body experience and autoscopy of neurological origin. Brain 127(Pt 2), 243–258 10.1093/brain/awh040 14662516

[B6] Blanke O. Mohr C. (2005). Out-of-body experience, heautoscopy, and autoscopic hallucination of neurological origin Implications for neurocognitive mechanisms of corporeal awareness and self-consciousness. Brain Res. Brain Res. Rev. 50, 184–199 10.1016/j.brainresrev.2005.05.008 16019077

[B7] Blanke O. Ortigue S. Landis T. Seeck M. (2002). Stimulating illusory own-body perceptions. Nature 419, 269–270 10.1038/419269a 12239558

[B8] Britton W. B. Bootzin R. R. (2004). Near-death experiences and the temporal lobe. Psychol. Sci. 15, 254–258 10.1111/j.0956-7976.2004.00661.x 15043643

[B9] Charland-Verville V. Bruno M. A. Bahri M. A. Demertzi A. Desseilles M. Chatelle C. (2013). Brain dead yet mind alive: a positron emission tomography case study of brain metabolism in Cotard's syndrome. Cortex 49, 1997–1999 10.1016/j.cortex.2013.03.003 23664000

[B10] Cobcroft M. D. Forsdick C. (1993). Awareness under anaesthesia: the patients' point of view. Anaesth. Intensive Care 21, 837–843 8122744 10.1177/0310057X9302100616

[B11] Els T. Kassubek J. Kubalek R. Klisch J. (2004). Diffusion-weighted MRI during early global cerebral hypoxia: a predictor for clinical outcome? Acta Neurol. Scand. 110, 361–367 10.1111/j.1600-0404.2004.00342.x 15527448

[B12] Facco E. Agrillo C. (2012). Near-death-like experiences without life-threatening conditions or brain disorders: a hypothesis from a case report. Front. Psychol. 3:490 10.3389/fpsyg.2012.00490 23162522 PMC3498963

[B13] French C. C. (2001). Dying to know the truth: visions of a dying brain, or false memories? Lancet 358, 2010–2011 10.1016/S0140-6736(01)07133-1 11755600

[B14] French C. C. (2005). Near-death experiences in cardiac arrest survivors, in Progress in Brain Research, Vol. 150, ed Laureys S. (Amsterdam: Elsevier), 351–367 10.1016/S0079-6123(05)50025-6 16186035

[B15] Gabbard G. O. Twemlow S. W. Jones F. C. (1981). Do “near death experiences” occur only near death? J. Nerv. Ment. Dis. 169, 374–377 10.1097/00005053-198106000-00006 7229635

[B16] Gabbard G. Twemlow S. (1991). Do “near-death experiences” occur only near-death?- Revisited. J. Near Death Stud. 10, 41–47 10.1007/BF01073295 7229635

[B17] Gallup G. Proctor W. (1982). Adventures in Immortality: a Look Beyond the Threshold of Death. New York, NY: McGraw-Hill

[B18] Greyson B. (1983). The near-death experience scale. Construction, reliability, and validity. J. Nerv. Ment. Dis. 171, 369–375 10.1097/00005053-198306000-00007 6854303

[B19] Greyson B. (1990). Near-death encounters with and without near-death experiences: comparative NDE Scale profiles. J. Near Death Stud. 8, 151–161 10.1007/BF01074000

[B20] Greyson B. (1998). Biological aspects of near-death experiences. Perspect. Biol. Med. 42, 14–32 9894355 10.1353/pbm.1998.0039

[B21] Greyson B. (2000). Near-death experiences, in Varieties of Anomalous Experiences: Examining the Scientific Evidence, eds Cardena E. Lynn S. Krippner S. (Washigton, DC: American Psychological Association), 315–352 10.1037/10371-010

[B22] Greyson B. (2003a). Incidence and correlates of near-death experiences in a cardiac care unit. Gen. Hosp. Psychiatry 25, 269–276 10.1016/S0163-8343(03)00042-2 12850659

[B23] Greyson B. (2003b). Near-death experiences in a psychiatric outpatient clinic population. Psychiatr. Serv. 54, 1649–1651 10.1176/appi.ps.54.12.1649 14645808

[B24] Greyson B. (2007). Consistency of near-death experience accounts over two decades: are reports embellished over time? Resuscitation 73, 407–411 10.1016/j.resuscitation.2006.10.013 17289247

[B25] Heim A. (1891). Notizen uber den Tod durch Absturtz. Jahrbuch des schweizer. Alpenclub 27, 327–337

[B26] Hoepner R. Labudda K. May T. W. Schoendienst M. Woermann F. G. Bien C. G. (2013). Ictal autoscopic phenomena and near death experiences: a study of five patients with ictal autoscopies. J. Neurol. 260, 742–749 10.1007/s00415-012-6689-x 23086176

[B27] Hou Y. Huang Q. Prakash R. Chaudhury S. (2013). Infrequent near death experiences in severe brain injury survivors - A quantitative and qualitative study. Ann. Indian Acad. Neurol. 16, 75–81 10.4103/0972-2327.107715 23661968 PMC3644787

[B28] Kelly E. W. (2001). Near-death experiences with reports of meeting deceased people. Death Stud. 25, 229–249 10.1080/07481180125967 11785541

[B29] Klemenc-Ketis Z. Kersnik J. Grmec S. (2010). The effect of carbon dioxide on near-death experiences in out-of-hospital cardiac arrest survivors: a prospective observational study. Crit. Care 14, R56 10.1186/cc8952 20377847 PMC2887177

[B30] Lai C. F. Kao T. W. Wu M. S. Chiang S. S. Chang C. H. Lu C. S. (2007). Impact of near-death experiences on dialysis patients: a multicenter collaborative study. Am. J. Kidney Dis. 50, 124–132, 132 e121–122. 10.1053/j.ajkd.2007.04.021 17591532

[B31] Lange R. Greyson B. Houran J. (2004). A Rasch scaling validation of a ‘core’ near-death experience. Br. J. Psychol. 95(Pt 2),161–177 10.1348/000712604773952403 15142300

[B32] Lempert T. Bauer M. Schmidt D. (1994). Syncope and near-death experience. Lancet 344, 829–830 10.1016/S0140-6736(94)92389-2 7916113

[B33] Lindley J. H. Bryan S. Conley B. (1981). Near-death experiences in a Pacific Northwest American population: the evergreen study. J. Near Death Stud. 1, 104–124

[B34] Lopez U. Forster A. Annoni J. M. Habre W. Iselin-Chaves I. A. (2006). Near-death experience in a boy undergoing uneventful elective surgery under general anesthesia. Paediatr. Anaesth. 16, 85–88 10.1111/j.1460-9592.2005.01607.x 16409537

[B35] Martens P. R. (1994). Near-death-experiences in out-of-hospital cardiac arrest survivors. Meaningful phenomena or just fantasy of death? Resuscitation 27, 171–175 10.1016/0300-9572(94)90010-8 8029538

[B36] McKay R. Cipolotti L. (2007). Attributional style in a case of Cotard delusion. Conscious. Cog. 16, 349–359 10.1016/j.concog.2006.06.001 16854594

[B37] Mobbs D. Watt C. (2011). There is nothing paranormal about near-death experiences: how neuroscience can explain seeing bright lights, meeting the dead, or being convinced you are one of them. Trends Cogn. Sci. 15, 447–449 10.1016/j.tics.2011.07.010 21852181

[B38] Moody R. A. (1975). Life After Life. New York, NY: Bantam Press

[B39] Nelson K. R. Mattingly M. Lee S. A. Schmitt F. A. (2006). Does the arousal system contribute to near death experience? Neurology 66, 1003–1009 10.1212/01.wnl.0000204296.15607.37 16606911

[B40] Nelson K. R. Mattingly M. Schmitt F. A. (2007). Out-of-body experience and arousal. Neurology 68, 794–795 10.1212/01.wnl.0000256784.85952.6f 17339596

[B41] Osterman J. E. Hopper J. Heran W. J. Keane T. M. van der Kolk B. A. (2001). Awareness under anesthesia and the development of posttraumatic stress disorder. Gen. Hosp. Psychiatry 23, 198–204 10.1016/S0163-8343(01)00142-6 11543846

[B42] Owens J. E. Cook E. W. Stevenson I. (1990). Features of “near-death experience” in relation to whether or not patients were near death. Lancet 336, 1175–1177 10.1016/0140-6736(90)92780-L 1978037

[B43] Parnia S. Waller D. G. Yeates R. Fenwick P. (2001). A qualitative and quantitative study of the incidence, features and aetiology of near death experiences in cardiac arrest survivors. Resuscitation 48, 149–156 10.1016/S0300-9572(00)00328-2 11426476

[B44] Posner J. B. Saper C. B. Plum F. (2007). Diagnosis of Stupor and Coma. New York, NY: Oxford University Press

[B44a] Ring K. (1980). Life at Death: A Scientific Investigation of the Near-Death Experience. New York, NY: Coward McCann & Geoghegan

[B45] Ring K. (1984). Heading Toward Omega: In Search of the Meaning of the Near-Death Experience. New York, NY: William Morrow

[B46] Rodin E. A. (1980). The reality of death experiences. A personal perspective. J. Nerv. Ment. Dis. 168, 259–263 10.1097/00005053-198005000-00001 7365486

[B47] Sabom M. (1982). Recollections of Death: a Medical Investigation. New York, NY: Harper & Row

[B48] Schwaninger J. Eisenberg P. Schechtman K. Weiss A. (2002). A prospective analysis of near-death experiences in cardiac arrest patients. J. Near Death Stud. 20, 215–232 10.1023/A:1015258818660

[B49] Stevenson I. Cook E. McClean-Rice N. (1989-1990). Are persons reporting “near-death experiences” really near death? A study of medical records. Omega 20, 45–54 17591532

[B50] van Lommel P. van Wees R. Meyers V. Elfferich I. (2001). Near-death experience in survivors of cardiac arrest: a prospective study in the Netherlands. Lancet 358, 2039–2045 10.1016/S0140-6736(01)07100-8 11755611

[B51] Woerlee G. M. (2005). Mortal Minds: the Biology of Near-Death Experiences. Amherst, NY: Prometheus Books

